# Psychological framework to understand interpersonal violence by forensic patients with psychosis

**DOI:** 10.1192/bjp.2023.132

**Published:** 2024-02

**Authors:** Sinéad Lambe, Kate Cooper, Seena Fazel, Daniel Freeman

**Affiliations:** Department of Experimental Psychology, University of Oxford, Oxford, UK; and Oxford Health NHS Foundation Trust, Oxford, UK; Department of Psychology, University of Bath, Bath, UK; Oxford Health NHS Foundation Trust, Oxford, UK; and Department of Psychiatry, University of Oxford, Oxford, UK

**Keywords:** Psychotic disorders/schizophrenia, forensic psychiatry, qualitative research, risk assessment, violence

## Abstract

**Background:**

Forensic patients with psychosis often engage in violent behaviour. There has been significant progress in understanding risk factors for violence, but identification of causal mechanisms of violence is limited.

**Aims:**

To develop a testable psychological framework explaining violence in psychosis – grounded in patient experience – to guide targeted treatment development.

**Method:**

We conducted in-depth interviews with 20 patients with psychosis using forensic psychiatric services across three regions in England. Interviews were analysed using reflexive thematic analysis. People with lived experience contributed to the analysis.

**Results:**

Analysis of interviews identified several psychological processes involved in the occurrence of violence. Violence was the dominant response mode to difficulties that was both habitual and underpinned by rules that engaged and justified an attack. Violence was triggered by a trio of sensitivities to other people: sensitivity to physical threat, from which violence protected; sensitivity to social disrespect, by which violence increased status; and sensitivity to unfairness, by which violence delivered revenge. Violence was an attempt to regulate difficult internal states: intense emotions were released through aggression and violence was an attempt to escape being overwhelmed by voices, visions or paranoia. There were different patterns of emphasis across these processes when explaining an individual participant's offending behaviour.

**Conclusions:**

The seven-factor model of violence derived from our analysis of patient accounts highlights multiple modifiable psychological processes that can plausibly lead to violence. The model can guide the research and development of targeted treatments to reduce violence by individuals with psychosis.

Approximately 60–70% of patients in forensic secure services in the UK have a primary diagnosis of psychosis.^1^ Typical offences include violent offences (e.g. assault, grievous bodily harm), attempted murder and manslaughter.^2^ There has been extensive research identifying those patients with psychosis at high risk of committing violence. Static risk factors such as previous criminal convictions, young age, male gender and a history of substance misuse have the largest associations with future violence.^3,4^ Dynamic risk factors such as hostile behaviour, recent drug or alcohol misuse, poor impulse control and non-adherence to psychological therapies or medication also predict violence.^4^ There is clear progress in understanding risk factors for violence, but the causal mechanisms driving violence are still largely unknown.

## Models of violence in psychosis

To date, a small number of psychological models have been proposed to explain violence by individuals with psychosis. On the basis of a narrative review, Lamsma & Harte^5^ and Volavka & Citrome^6^ propose different causal pathways between predisposing risk factors (including social factors such as family history of criminality or a neighbourhood with social problems), psychiatric disorder (e.g. schizophrenia, comorbid antisocial personality disorder, substance misuse), psychiatric symptoms (e.g. hostility, positive symptoms, agitation) and precipitating factors (e.g. stress, treatment non-adherence). Other models have focused on a single driving factor for violence. Adams & Yanos^7^ proposed that violence in psychosis is primarily driven by anger, exacerbated by substance misuse and impulsivity. Anger is hypothesised to arise from a number of factors, including victimisation, social stressors, anxious arousal and hostile attribution bias. Two theories suggest that specific psychosis symptoms lead to violence.^8,9^ The threat/control-override theory posits that violence occurs when delusions cause a person to feel personally threatened or involve the intrusion of thoughts that override self-control;^8^ this has not been consistently replicated.^10^ The second theory is that command hallucinations, when the person believes the voices are omnipotent and powerful, result in violence.^9^ This has led to treatments designed to reduce compliance with command hallucinations.^11^

## Treatments

Clozapine is currently the most effective pharmacological treatment for aggressive and violent behaviour in people with psychosis.^12,13^ However, the effects of clozapine are unspecific and the underlying mechanism is not yet understood.^14^ The available evidence on psychological interventions is more limited. Few randomised controlled trials have been conducted of psychological interventions targeting the reduction of violence by forensic patients with psychosis. Trials have tested dialectical behaviour therapy,^15^ reasoning and rehabilitation,^16^ cognitive remediation,^17^ cognitive–behavioural therapy for psychosis with anger management,^18^ and virtual reality aggression prevention therapy.^19^ Findings of these have been mixed (e.g. change in one domain of violence, such as verbal aggression, but not in others)^16,18,19^ or difficult to interpret owing to small numbers^15^ or high levels of attrition.^16^ The development of effective psychological therapy requires a theoretically driven approach. Treatment techniques need to target key mechanisms that contribute to the occurrence of aggression.^20,21^ Our understanding of these mechanisms could be advanced by speaking with the patients themselves. Acceptability of a treatment is likely to be enhanced if models are aligned with patient experience.

## The present study

To date, there has been no qualitative study of psychological factors leading to violence in patients with psychosis. The present study aimed to understand, from forensic patients with psychosis, the key psychological processes that may drive violent behaviour. We are focusing on violence in people with psychosis for several reasons: (a) there is an elevated risk of violent offending, (b) there is typically contact with health services before and after the offence, providing an opportunity for both prevention and intervention, and (c) there are clinical features specific to this group (e.g. positive symptoms) that need to be accounted for in any model and subsequent treatment. For this study we defined violence and aggression as the intentional use of physical force or power, threatened or actual, against another person that either results in or has a high likelihood of resulting in injury, death or psychological harm. Studies have found that the risk factors for minor and more severe forms of interpersonal violence are similar,^4^ thus participants were interviewed about a range of violent incidents, including their index offences.

## Method

### Study design

We used reflexive thematic analysis^22,23^ to investigate the psychological processes involved in violence by forensic patients with psychosis. Reflexive thematic analysis is a qualitative research method for analysing and interpreting patterns across a data-set of interviews. It requires researchers to critically interrogate their own experiences, preconceptions and biases and consider how these may affect the analytical process. We took a critical realist approach to the analysis. Critical realism theory posits that there exists an objectively knowable reality, but acknowledges that perception and cognition influence how that reality is observed.

### Participants

Purposive sampling was used to ensure representation across stages of recovery and severity and form of violent behaviour. A sample of 20 participants allowed for in-depth interviewing and analysis while also ensuring a sufficient breadth of viewpoints. Participants were recruited from three National Health Service (NHS) trusts: Oxford Health NHS Foundation Trust (Oxford, UK), West London NHS Trust (London, UK) and Devon Partnership NHS Trust (Devon, UK). Participants were included in the study if they were over 18 years old, identified as male, and had a primary diagnosis of schizophrenia spectrum disorder and a history of interpersonal violence. Participants were excluded if their offending was primarily sexual violence, if they had an intellectual disability or if their verbal English was insufficient to take part in the interview.

### Procedure

Participants were initially told about the research by their clinical team and only if they expressed an interest were they approached by the research team. Limitations of confidentiality were clearly explained. Participants provided written informed consent to take part and for quotes to be used under a pseudonym in later publications. Participants were given a 2 week cooling-off period, when they could contact the researcher to withdraw and have their interview deleted.

All interviews were conducted by a clinical psychologist (S.L.) trained in reflexive thematic analysis. Interviews were audio-recorded and later transcribed verbatim by a professional transcription service. Interviews were semi-structured to facilitate a more open and flexible discussion and to allow the exploration of unanticipated themes. The interview guide (Supplementary material 1, available at https://dx.doi.org/10.1192/bjp.2023.132) was developed based on input from a lived experience advisory panel (four people who had had psychosis and used forensic psychiatric services), discussions with clinicians working in forensic services and a review of literature on risk factors for violence by forensic patients with psychosis. Three pilot interviews were conducted with people with lived experience to ensure the questions and interview style promoted openness and elicited the depth of description needed to achieve the study aim. These data were not included in the analysis.

Interviews started with an initial open question about the participant's experience with violence. Subsequent questions focused on the initial emergence of violent behaviour, an in-depth description of significant incidents of committing violence, including the build-up to the incidents, and the thoughts and emotions before, during and after. Questions also explored ‘near misses’, when there was an urge to be violent that was not acted on, which aimed to identify inhibiting factors for violence and understand how participants experienced the consequences of not being violent when feeling provoked. It was emphasised that the information given by the participant in the interview would be shared with care teams only if a disclosure significantly altered their current risk assessment. Follow-up questions and probes were used as appropriate. Interviews were transcribed verbatim, anonymised and checked for accuracy.

The authors assert that all procedures contributing to this work comply with the ethical standards of the relevant national and institutional committees on human experimentation and with the Helsinki Declaration of 1975, as revised in 2008. The study received approval from an NHS research ethics committee (London – Riverside Research Ethics Committee; ref 21/LO/0892).

### Analysis

The analysis followed the guidance provided by Braun & Clarke^22,23^ for reflexive thematic analysis (for more details on the analytical process see Supplementary material 2). Audio recordings were listened to and transcriptions read and re-read to ensure familiarity. All transcripts were coded by S.L., paying attention to both descriptive and conceptual elements within the data. Codes were considered against the whole data-set and similar codes combined. Thematic maps were used to aid the development of themes and consider the links and relationships between themes. Themes were considered in relation to the research aim and any themes that fell outside the study remit or did not have sufficient data to support them were discarded. Quotations were selected if they provided a good summary of the core of a theme and represented views across the range of participants. Codes and themes were reviewed in supervision (D.F., S.F. and K.C.) and by members of the lived experience advisory panel to ensure that a rigorous credibility analysis was conducted.^24^

## Results

Recruitment took place from 23 February 2022 to 24 May 2022. Of 22 patients approached, 20 consented to take part in the interview. Participants’ mean age was 36.4 years (s.d. = 11.0: range 20–56) and ethnicity was reported as White British (*n* = 10; 50%), White Other (*n* = 3), Black British (*n* = 2), White and Black Caribbean (*n* = 2), Black African (*n* = 1), White and Black African (*n* = 1) and Mixed White and Asian (*n* = 1). Participants’ index offences included violent offences such as assault, battery, grievous bodily harm, affray or use of an offensive weapon (*n* = 15), attempted murder (*n* = 2), manslaughter (*n* = 2) and murder (*n* = 1). Clinical and crime-related information is shown in Table 1.

**Table 1:** Participants’ clinical and offending information

	*n* (%)
Primary diagnosis	
Schizophrenia	17 (85)
Schizoaffective disorder	3 (15)
Secondary diagnoses	
None	11 (55)
Personality disorder (antisocial, borderline)	4 (20)
Substance misuse	4 (20)
Generalised anxiety disorder	1 (5)
Attention-deficit hyperactivity disorder	1 (5)
Forensic service	
Medium secure acute ward	3 (15)
Medium secure rehab ward	14 (70)
Low secure ward	2 (10)
Community forensic service	1 (5)
Previously admission to high secure forensic unit	1 (5)
Previously admission to medium secure forensic unit	9 (45)
Previous convictions	13 (65)
Previous violent conviction	11 (55)
	Mean (s.d.)
Length of forensic admission (months)	33.8 (28.2)

The analysis identified three themes: (1) Violence as the dominant response mode; (2) Violence protects against a trio of sensitivities to other people; and (3) Violence relieves negative internal states. There were seven subthemes across these three themes, each representing a different psychological factor contributing to violence (Appendices 1 and 2).

### Theme 1: Violence as the dominant response mode

In this theme, violence had become the dominant response to difficulties. It has become an established behaviour through social learning (subtheme 1.1: Violence is all I know). Participants had a rigid set of rules, which guided their behaviour during a confrontation (subtheme 1.2: Rules of engagement). These rules provided a way for participants to justify their behaviour.

#### Subtheme 1.1: Violence is all I know

Violence was a habitual response to interpersonal difficulties and happened without significant conscious thought:

‘Just brushing past me or stand next to me. I just turn around and smack them.’ (Kyle)

Family members and peers modelled and normalised the use of violence. Participants were exposed to violence, criminality and drugs as they grew up. Local criminal gangs often displayed wealth and this lifestyle was further promoted in favoured music and films. In this context violence achieved results, was reinforced by peers and formed part of a positive social identity:

‘It becomes so normal to you that you don't even see it as aggressive behaviour.’ (Amadi)

‘My older brother, he's six years older than me, he was a football hooligan, and he liked fighting, and he was an influence on me. He was always wrestling me, telling me he was going to toughen me up, and I should fight at school and be the hardest, and, constantly telling me that. I grew up thinking that. I grew up thinking I had to be big and hard and fight people.’ (Dean)

‘I could have started saying look, I'm a bad man, I'm a gangster, who the fuck are you, fuck… do you know who you're messing with? Who do you think you're talking to?’ (Juan)

A limited behavioural repertoire for managing interpersonal difficulties meant that even when motivated, participants struggled to conceive how situations could be handled without violence, beyond the complete avoidance of triggers:

‘I don't have a choice, some people would say there's always a choice but, I don't think I'll ever be normal again, like I'd love to be. I'd feel uncomfortable going out on dates with girls because of things that could arise. Like going out with a girl, going to a bar, there's a situation when I'm drunk, someone is trying to chat to my girl. So I'm saying “That, that's my girl. Don't chat to her”, and then if he gets lairy, I'll end up bottling the guy.’ (Jamar)

#### Subtheme 1.2: Rules of engagement

Rules guided behaviour during a violent confrontation with the aim of increasing the chance of winning the fight, maintaining a reputation and providing justification. Key rules included: if you believe someone is going to harm you, it is best to hit them first, hit them until they can't get up and scare them enough that they won't retaliate in future:

‘Because I don't want to get attacked first, I don't want to get hit first, so I always attack first.’ (Jamar)

‘But say if they see me, that's when I pull out my weapon, I will try and chase them or try stabbing them or something. Because I want to scare them. Or else I'll probably get stabbed or something, yes or it will happen to me.’ (Adam)

Some rules reflected the views of wider social networks on how to behave during a confrontation. For example, if a friend is in a fight then you must step in too; if someone brings trouble to you, you must deal with it rather than involving the police. These rules led to more reactive and extreme violence and perpetuated cycles of violence:

‘If you see your friend get scared, well, now you've got to step up because he's part of your group of people, so now you've got to let the people know they can't mess with your mate like that because he's got back up.’ (Dan)

‘I didn't press charges against the guy that stabbed me.’ (Juan)

The threshold at which violence was considered a justified response was low, whereas the degree of force deemed appropriate was severe. It was often black and white – if someone was considered to have ‘crossed a line’ then any level of violence was justified. These justifications allowed absolution of guilt or remorse:

‘And I walked up to him and I was like… like “You say that again to my face”. And he said it again to my face. I grabbed him. I punched him until he was a puddle. I punched him really really hard on the face over and over again.’ (Lewis)

‘I wouldn't feel anything. I wouldn't feel remorse. I just carry on what I'm doing. I won't give a shit. They tried something with me and I put them in their place and that's the way it was. That's the way me and my mates score up.’ (Kyle)

### Theme 2: Violence protects against a trio of sensitivities

In this theme, violence was a response to the behaviour of others. Participants were highly sensitive to disrespect (subtheme 2.1), threats of physical harm (subtheme 2.2) and unfair treatment (subtheme 2.3), whether real or imagined. They held positive beliefs about violence as a way of increasing social status, protecting oneself and getting justice.

#### Subtheme 2.1: Sensitivity to disrespect

Subtle behaviours, such as body language or tone of voice, were interpreted as disrespectful. Violence was used to re-establish social status. Delusional content (e.g. that others were laughing at or mocking them) amplified this process:

‘Look. Like, if you talk to me in a certain way and I don't like it, I'm gonna fight you.’ (Amadi)

‘My index offence is somebody that was in supported lodging that I believed was reading my mind and yeah I stabbed him with a knife. Yeah, mocking me, laughing at me. Knew exactly what I was thinking.’ (Dean)

Participants described feeling ‘worthless’ and ‘unloved’ from growing up in poverty and being bullied or criticised. Violence became an effective way for them to prove themselves and gaining respect:

‘My other brother, who was six years older, always bullied me and tried to humiliate me and mock me in front of people.’ (Dean)

‘Because your life is worthless, you don't mind if you hurt somebody else's life, so that aggressiveness can come out of you from time to time.’ (Amadi)

‘I had my first fight when I was about eight, nine years old. And then I found out what it was like for people to be frightened of me and respect me. And then things just escalated from there.’ (Juan)

In contrast, walking away from a fight reduced participants’ sense of social status and often triggered angry rumination:

‘In the past I wouldn't [walk away from a fight] because they'd just take you for an idiot. Other people would be saying “Oh, he's an idiot”.’ (Sorin)

#### Subtheme 2.2: Sensitivity to physical harm

Participants were vigilant for signs of threat and would respond with force:

‘I'm always on guard, I'm always thinking, threat, threat, threat, all the time. […] Shoulders, arms, body language, how they're moving and, like, how they're talking, tone of voice. You know something is about to kick off.’ (Jamar)

‘Anyone acknowledges that if they feel threatened, then they will get violent. It's like when people are paranoid, and they perceive people as a threat, they will act on it, and do something. Fight or flight mode, or they'll run away or they'll attack someone.’ (Dean)

Participants had been victims of extreme violence in the past and believed using violence was essential for protecting oneself. They often had examples of when using violence protected them or a member of their family. In contrast, being non-violent left them feeling more vulnerable to attack:

‘I've been attacked badly in the past, nearly killed. Jumped by eight people and they nearly kicked me to death. When I did nothing wrong.’ (Scott)

‘It was only till I was older and I started fighting back that he stop beating her [my mum].’ (Garry)

‘You get beaten up, get really hurt. If you don't fight back then people just walk all over you.’ (Kyle)

Psychosis triggered or amplified the sense of threat, leading to the use of violence. For some participants there was an interaction between real-world threats and paranoia. Participants described voices that were threatening or would warn of imminent threats from others. A number of participants’ attempts to explain to themselves the onset of psychosis led them to believe they were being poisoned. The use of violence was driven by the belief that it was the best means of protecting oneself and others:

‘I was smoking weed and I started hearing things. For example saying “Tommy is gonna kill ya, you'll have to get him”. I'll go and get him, I'll go the second I can, I thought they were coming for me so I went for them.’ (Garry)

‘I heard it with my ears. It said “Your neighbour's raping his girlfriend”. I heard it a few times and about a week before it happened. So, I went over with a hammer and I got in through his window.’ (Sam)

#### Subtheme 2.3: Sensitivity to unfairness

Participants had rigid ideas of how they should be treated and were sensitive to unfair treatment. The frustrations they faced were often interpreted as intentional attempts to upset them:

‘When I feel like other people are violating the terms of what they're supposed to do… I used to get angry quite a lot, especially with staff. Because I used to feel like they weren't treating me the way they were supposed to be treating me.’ (Amadi)

Participants described ruminating about reasons why they had been treated unfairly or injustices that they had suffered in the past. There was a strong desire to restore equilibrium through violence:

‘I really want to go and get revenge. I always be thinking about it and it got to a point where my girlfriend was like “You got to stop talking about it; it was like a year ago and still going on about it”.’ (Kyle)

‘If somebody accidentally hit me in the face with a piece of wood, I'd want to slap them back, even if, even if it was an accident. I don't think it would be completely out of order. They caused me pain then, I think I would give them some pain back.’ (Mateo)

This sense of unfairness was generalised by the participants to society as a whole. For example, two unprovoked attacks on strangers in the street by participants came from the belief that others had been dealt a better hand in life. Jealousy and resentment led to the use of violence:

‘Something that happened that day, I spotted, maybe spotted some loving couple holding their hands together getting inside of a rich car, Mercedes let's say … fancy car, and I thought they looked happy and I thought … and in comparison … I instantly compared myself to them where I thought that they were happy and I wasn't happy so it built up my anger.’ (Tomasz)

### Theme 3: Violence relieves negative internal states

In this theme, violence was a response to internal states. Violence had an emotion regulation function and was triggered by psychological arousal and strong emotions (subtheme 3.1: Physiological drive). Violence could also be a desperate attempt to escape or stop voices, visions and paranoia (subtheme 3.2: Escape from psychotic experiences).

#### Subtheme 3.1: Physiological drive

Violence was a response to intense feelings of anger or anxiety and provided an effective way for the individual to regulate difficult emotions:

‘Sometimes there's a pressure on the lungs. Your chest feels tight, your legs feel like jelly. Yeah, it's like you know when you get, what do they call it, your stomach churns. And you get pains in your arm, and then you let go, you kick off and it goes away. Anxiety, that's what they call it. That's it yeah, feels like I'm having a heart attack. And once you lash out with anger, it's released, that anxiety, the pressure.’ (Sorin)

‘I used to punch walls a lot, and my hands felt bad because my knuckles were all pushed in because I'd hit walls when I'm angry. I hit people. It was just a release and it hurts afterwards and it feel good like it takes it away.’ (Kyle)

In these cases the victim often had done nothing to trigger the perpetrator or had done something minor that had triggered a disproportionate response:

‘Just to hit somebody, random people were just being hit because I wanted to let the anger out and I didn't decide to punch a tree, I didn't decide to punch a stop sign on the way. I decided to punch somebody.’ (Tomasz)

Participants described being very reactive to even minor triggers such as repetitive noises. Poor inhibitory control when angry or anxious often led to violence:

‘I still get angry, very, very… bad anger. It comes up quickly, you know so it's hard to manage.’ (Pasquale)

‘There'd be no thoughts, I'd kick the shit out of them and they would take me to seclusion.’ (Garry)

There was a lack of knowledge of how to manage frustrations or difficult emotions without violence. There were also limited opportunities for practising alternative ways of dealing with anger:

‘I'll say, “Right, next time I'll try again, next time” and it's an ongoing thing, you can't practise all these skills until you're angry and it's hard to… to do everything right when you're angry.’ (Pasquale)

#### Subtheme 3.2: Escape from psychotic experiences

Violence was sometimes an act of desperation. Participants described terrifying experiences during an acute psychotic phase. They were overwhelmed by voices, visual hallucinations or perceptual distortions. Voices encouraged violence as a way to make these experiences stop. Violence was seen as a last resort to escape these experiences or to prevent family members from witnessing them:

‘I was freaking out. I was seeing people change into reptilian, shapeshifting aliens. I used to have conspiracy theories about it and it just got to my head. I started seeing people change in front of my eyes. I was a hundred percent sure it was real. I was too scared to talk about it. I was terrified … I thought if I did what I did, it would all stop. That's what the devil chant and voices were telling me to do this, it will stop. The moment I done it [stabbed someone] it just went quiet. Everything just went really quiet. The sun went in and I was like, this isn't going to stop, and I just walked out.’ (Kyle)

## Discussion

In this study we used qualitative methods to understand key psychological processes that may drive violent behaviour by people with psychosis. We conducted in-depth interviews with 20 patients with psychosis recruited from forensic psychiatric services in three regions of the UK. The rich descriptions shared by participants provided an insight into how they think about violence, the functions it serves and the perceived barriers to using alternative non-violent behaviours. Such insights can help orient clinicians to the patient perspective, allowing better calibration of clinical discussions about violence. Our analysis identified seven key psychological factors that contribute to the use of violence (Appendix 2). Violence is a learned behaviour, which can be both habitual (Factor 1) and underpinned by rules that engaged and justified an attack (Factor 2). Violence is often a protective strategy against a trio of sensitivities to other people: sensitivity to physical threat, from which violence protects (Factor 3); sensitivity to social disrespect, whereby violence increased status (Factor 4); and sensitivity to unfairness, whereby violence delivers revenge (Factor 5). Violence can have an emotion regulation function, whereby intense emotions are released (Factor 6), and violence can be an attempt to escape or gain control when overwhelmed by voices, visions and paranoia (Factor 7). The pattern of emphasis across these processes differs when explaining an individual patient's offending behaviour. This seven-factor framework is consistent with, and expands on, existing literature on violence in psychosis and provides multiple testable hypotheses about the underlying causal mechanisms. The multifactorial nature of the model reflects the complex nature of violence by people with psychosis. It can be used to distinguish between different patterns of violence, allowing for more individualised approaches to understanding and treating violence. Furthermore, it identifies potentially modifiable factors (habitual behaviour patterns, pro-violence beliefs and attitudes, signs of low tolerance of threat, social disrespect and injustice, poor emotion regulation and positive psychotic symptoms) and can be used to guide the development of new targeted interventions to reduce violence – an area that needs renewed attention.

### Risk factors and mediators of violence

#### Learned behaviour

Through social learning processes violence can become an established part of one's behavioural repertoire.^25^ Factors such as parental involvement in crime, early experiences of physical violence and violent victimisation are known risk factors.^4^ Many participants were exposed to crime and violence from a young age, with violence being normalised or even glorified. At times this violence was extreme in nature (e.g. chased with knives, stabbings). It could be argued that such environments not only teach but at times necessitate violence. In this context pro-criminal beliefs and maladaptive rules and assumptions about violence develop. Such beliefs have been indicted in violence in the general population^26^ but have not been explored as a mediator of violence in psychosis. Violent behaviour becomes habitual through use, with past violent behaviour being one of the strongest predictors of future violence.^4^ Any future treatment should have a strong behavioural component (e.g. role-plays, behavioural experiments) to establish and strengthen competing prosocial behavioural responses to difficult situations.

#### Sensitivity to others’ behaviour

Violence was often a response to other people's behaviour – triggered by the perception that others were being threatening, disrespectful or unfair. Similar to previous studies,^4^ experiences of violent victimisation, bullying, neglect, abuse and social injustice were common. These increased hypervigilance for threat and hostile attribution biases, further amplified by psychosis symptoms (e.g. voices or paranoia). Hypervigilance and persecutory beliefs are common in psychosis and in most cases does not lead to violence. However, it was the combination of perceived threat and the belief that violence was an effective method of protecting oneself, gaining respect or justice that led to violent behaviour. The use of violence also prevented opportunities for the correction of misinterpretations (e.g. they are staring at me because they are going to attack) and positive beliefs about violence (e.g. violence keeps me safe). Violence elicited aggression from others, reinforcing the view that others are threatening. The non-occurrence of an attack was misattributed to having acted aggressively and thus successfully scaring the person off. Consequently, the belief that violence is necessary persisted. Treatments should incorporate techniques to test out and correct these misinterpretations, build a tolerance for the behaviour of others and help patients develop prosocial ways of achieving safety, social respect and justice to reduce their reliance on violence.

#### Emotion regulation

Studies have shown anger to be an important driver of violence in psychosis.^7,27^ However, the influence of emotion regulation has not been explored, although there is some evidence it might play a role in violence in the general population.^28^ Violence did appear to have an emotion regulation function. Participants described high emotional reactivity and poor ability to regulate their response. Violence was effective in releasing intense feelings of anger or anxiety. The descriptions provided of violence releasing or taking away difficult feelings are reminiscent of self-harm literature, in which some acts are performed without suicidal intent but in order to regulate intense emotional states.^29^ Self-harm and violence frequently co-occur and the presence of one has been shown to increase the risk of the other.^24^ Difficulties in emotion regulation, which have been implicated in self-harm,^30^ may also play a role in violence. Future treatments could benefit from incorporating the development of emotion regulation skills.

#### Positive psychotic symptoms

For most participants positive psychotic symptoms, particularly paranoia and voices, contributed to violence by amplifying the aforementioned processes (sensitivity to others and emotion dysregulation). This is consistent with empirical findings that positive symptoms alone are a weak predictor of future violence.^4^ However, although less common in the study's participant group, violence was sometimes a direct a response to being overwhelmed by delusions, voices and perceptual distortions, and was a desperate attempt to escape these experiences or make them stop. In two accounts, violence occurred during their first episode of psychosis, was ego dystonic and had occurred within 24 h of contact with mental health services.

#### Substance misuse

Finally, it is important to comment on the role of substance misuse, as this is one of the strongest predictors of violence in psychosis.^3,31^ Surprisingly, no participants described substances as being a direct single cause of their violence, but rather substance use contributed across a number of the different factors. Involvement in taking drugs or drug dealing was an initial route into crime for several participants and led to some of the more extreme experiences of violence (e.g. between drug gangs). Alcohol and drug use increased anger and impulsivity. For some participants substances were used to relax and manage difficult feelings (emotion regulation) and agitation increased when going through withdrawals. Drug use also triggered or amplified psychosis symptoms such as voices and paranoia. Management of substance misuse should be included in any future treatment.

### Strengths and limitations

To our knowledge, this is the first qualitative study to explore the psychological processes of violence in psychosis. However, there are a number of study limitations. The nature of qualitative research means the sample is small and not representative. Therefore it is exploratory and the findings need to be tested in larger, more varied samples. In addition, it may be that those willing to participate in the current study differ from the general forensic population, for example in their ability to reflect on their violence. However, there was a high uptake, with only two people approached about the study declining to take part. This work focused on violence by people with psychosis, which suggests that violence by such individuals differs in nature from that of the general population. However, this may not be the case and some elements of this model may have wider applicability. The study is reliant on retrospective accounts, which may not always be accurate. In addition, violence is a stigmatised issue and, although efforts were taken to manage this, participants may not have been completely open. However, the detailed descriptions of violence provided some assurances that this was not a significant problem. People with lived experience were involved in the study design and analysis; however, more participatory methods (e.g. interviews conducted by peer researchers) could have been used to enrich the analytical process and should be considered in future research.

## Supporting information

Lambe et al. supplementary material 1 Lambe et al. supplementary material

Lambe et al. supplementary material 2 Lambe et al. supplementary material

## Data Availability

The data are not publicly available because they contain information that could compromise the privacy of research participants. The data may be made available from the corresponding author, S.L., on reasonable request.

## Appendix 1

### Three themes and seven subthemes illustrated by quotations

**Table:**

**Theme 1: Violence is the dominant response mode**
Subtheme 1.1: Violence is all I know
‘I grew up with violence, I grew up with gangs. Violence is all I know.’ (Garry)
‘If you think like people are lying to you then that [violence] is the only way deal with it.’ (Sorin)
Subtheme 1.2: Rules of engagement
‘You know someone's saying something bad and talking shit about you, you go and deal with it. I lived by these rules for a long time.’ (Kyle)
‘Something could happen to me, so I don't stop until I know they're out.’ (Jamar)
‘I mean, once I've beaten them up and they're lying on the floor and they're bleeding or they're unconscious I will stop; I won't kill them.’ (Pasquale)
‘Sometimes like my friend will get in trouble and I have to fight because he's in trouble.’ (Adam)
**Theme 2: Violence protects against a trio of sensitivities**
Subtheme 2.1: Sensitivity to disrespect
‘I was not respected at home. I was not loved, you know, and I was abused. So I felt better when I was in the gang, I thought “Well, I am somebody now”, do you know what I'm saying, even though I'm a criminal.’ (Pasquale)
‘All I want to do is fight. I always have to prove myself or if I think someone's better than me or something being violent is the way forward.’ (Kyle)
‘When I see someone back down from something, I think “Oh, he's now accepted a lower rank in the hierarchy than another person who's now taking his place in the ranking of the hierarchy”. There are some people who stay completely out of it, and I struggle to understand how they do it.’ (Mark)
‘I was travelling on the bus, I thought people had taken the mick out of me, so I had a bit of a go at them. They were just sat really close to me and just laughing all the time. Thought they're probably making fun of me.’ (Scott)
Subtheme 2.2: Sensitivity to physical harm
‘Punches. Crutches hit across my head, iron bars hit across my head. You know I got stabbed when I was 17. I was in a coma.’ (Juan)
‘I became a scary person in, like, year seven, like no one would, like, fuck with me anymore because I'd, like, asserted my myself with violence.’ (Lewis)
‘You didn't punch them back, but in my mind I'm thinking “Yes, maybe I did the right thing but it's still making me feel scared, I still feel scared and anxious now, they'll continue to do that until I punch them”.’ (Pasquale)
‘When I got stabbed, I kept calling the police, said I wanted police protection. I didn't press charges against the guy that stabbed me. But I kept thinking he was going to come back and finish me off. But that's the time when my schizophrenia started.’ (Juan)
‘I used to fight the wardens a lot in prison and I used to think it was them putting things in my food to make me hear the voices.’ (Pasquale)
Subtheme 2.3: Sensitivity to unfairness
‘They do want to frustrate me, yeah obviously they, they know it frustrates me, makes me angry, I but I don't know why they want to do that.’ (Dean)
‘Even if I killed him. Even if it takes like ten years, I have to get him back. Like I've got a couple of people like that, like’ if I see him, yes, I have to get him back, I have to get him back. I don't know why. Every time I think about it, it gets… every time I think about this it gets me really angry. Like I get really agitated and stuff.’ (Adam)
‘And I was watching a YouTube video, I remember with (Dan) Bilzerian, he has more than me and he had a lot of things and I couldn't even get 20 quid on my credit card. So, I start thinking what use is my life right here. He's got everything and I got nothing, too different, too different. It killed me inside. I wanted to take his life.’ (Reece)
**Theme 3: Violence relieves negative internal states**
Subtheme 3.1: Physiological drive
‘It's like a, like a pressure but when you kick off it releases, relieves stress, so when you kick off you feel much better.’ (Sorin)
‘Anger can be released in a wrong way, you can see anger as a ticking bomb, as a ticking bomb inside of me. It can detonate. It can explode in the violence and aggression. Well, it happened to me on five occasions where I hit someone in the face because of my anger.’ (Tomasz)
Subtheme 3.2: Escape from psychotic experiences
‘I thought that a crisis team were taking my kids away to mutilate them and bring them back with all their guts hanging out and everything to show my wife and my dad and that's why I attacked my wife and my dad so that's it. Where's that come from I've got no idea, how that fed into violence I mean it's just ridiculous, it doesn't make any sense I can't put my finger on it, I just got no idea I just didn't want them to see my kid mangled, but it doesn't make any sense. But that was it but what turned it into violence, is I just didn't want my wife or dad to see my mutilated kids.’ (Jack)

## Appendix 2

### Seven-factor model of violence in psychosis

**Table:**

**Violence as the dominant response mode**
Factor 1: Violence is all I know Violence modelled, normalised and reinforced by social network Reinforced by proceeds of crime and drug dealing Practice has ingrained behaviour Forms part of positive social identity Deficit in prosocial problem-solving skills
Factor 2: Rules of engagement If there is going to be a fight, make sure you throw the first punch Hurt them enough that they won't retaliate If your friend is in a fight, you have to get involved You deal with problems yourself, you don't involve the police If they cross a line, any level of violence is justified (black and white thinking)
**Violence protects against a trio of sensitivities**
Factor 3: Sensitivity to threat of physical harm Experience as victim of extreme violence Hypervigilance for signs of threat Misinterpretation others’ behaviour (e.g. eye contact) as threatening Triggered or reinforced by paranoia and voices. Memories of violence protecting self and others from harm Belief that violence is protective and not using violence (i.e. prosocial response) is dangerous
Factor 4: Sensitivity to social disrespect Experiences of neglect, bullying, emotional abuse Low self-worth Hypervigilance for signs of disrespect Misinterpretation of others’ behaviour as disrespectful or belittling Violence provides sense of self-esteem and status Others being fearful is interpreted as respect Belief that walking away from a fight is degrading
Factor 5: Sensitivity to unfairness Frustrations interpreted as intentional injustices by others Rumination about being treated unjustly Build-up of resentment towards others Revenge drive
**Violence relieves difficult internal states**
Factor 6: Physiological drive Heightened anxiety and/or anger Emotionally reactive Poor emotion regulation skills Violence relieves intense feeling of anger or anxiety
Factor 7: Escape from psychosis experiences Intense experiences of voices, visual hallucinations and perceptual distortions High levels of belief and absorption in positive symptoms High levels of distress Delusional experiences support use of violence (e.g. voices encouraging) Violence seen as a last option to stop or escape experiences
