# Supplementary material for: Psychological framework to understand interpersonal violence by forensic patients with psychosis
Source: Br J Psychiatry. 2024 Feb;224(2):47–54. doi: 10.1192/bjp.2023.132 (PMC10807973; doi:10.1192/bjp.2023.132)
Supplement: Lambe et al. supplementary material 1 — Lambe et al. supplementary material [file S0007125023001320sup001.docx]

**Supplementary materials 1: The interview guide**

**Topic guide**

Note: these questions were a guide for the interview. The interview was semi-structured to respond to and explore the what is said by the participant.

What has been your experience with aggression and violence?

When did you first start using violence?

What kinds of situations have led to violence for you?

Can you talk me through a situations that stands out in your mind? /a typical time / a worst case

What was going on for you at the time?

What were you thinking? Before, during, after

What were you feeling? Before, during, after

How did others react?

What was the outcome?

How do you think about it now?

What leads to you using violence? What things contribute to you being violence?

Impact of substances?

Impact of mental health?

Have there been times you have wanted to be violent and didn’t?

What was going on for you at the time?

What were you thinking? Before, during, after

What were you feeling? Before, during, after

How did others react?

What was the outcome?

How do you think about it now?

Is there anything else I haven’t asked about that I should have?

Is there anything else I would need to know to better understand violence?
