# Supplementary material for: Psychological framework to understand interpersonal violence by forensic patients with psychosis
Source: Br J Psychiatry. 2024 Feb;224(2):47–54. doi: 10.1192/bjp.2023.132 (PMC10807973; doi:10.1192/bjp.2023.132)
Supplement: Lambe et al. supplementary material 2 — Lambe et al. supplementary material [file S0007125023001320sup002.docx]

**Supplementary materials 2: Positionality and reflexivity.**

Critical realism theory posits that there exists an objectively knowable reality, but acknowledges that perception and cognition influence how that reality is observed. In other words there is a singular true reality but it cannot be accessed directly, rather the beliefs and experiences of the researcher influences how that reality is perceived, interpreted and represented. It is therefore necessary for the researchers to critically interrogate their own experiences, preconceptions, and biases and consider how these may affect the analytic process. This aligns with reflexive thematic analysis, which emphasised the importance of reflexivity throughout the analytic process. I will therefore describe my positionality, as well as that of my supervisors, and then describe how this self-awareness contributed to a reflexive research practice.

I am a female clinical psychologist and work in the National Health Service (NHS) as well as conducting research at the University of Oxford. My clinical work focusses on cognitive behaviour approaches to treating psychosis and I have worked in for general adult mental health services and forensic mental health services. I am supported by a supervisory team specialising in cognitive approaches to psychosis (DF), forensic psychiatry (SF), and qualitative research methods (KC). The research was also supported by lived experience advisory panel (LEAP) who had difficulties with interpersonal violence and had been admitted to medium secure forensic unit. At the onset of the study SL had discussions with each member of the research team about their perspectives on the nature of violence in psychosis.

As a clinical psychologist with an interest in cognitive behaviour therapy, I was aware that I would be drawn to developmental and psychological factors that lead to violence. I was therefore careful to listen attentively to accounts of other factors, for example related to biological factors or psychopathology. I checked out my interpretations with the LEAP and my supervision team – supervision with a forensic psychiatrist was particularly helpful for interrogating my interpretation.

I have no direct exposure to violence though I had worked with many perpetrators and victims of violence in my work as a clinical psychologist. Listening to accounts from participants was often like entering a different world. I therefore tried to explore events and topics raised in a very open and neutral way. I tried to not make any assumptions about what these experiences would be like (instead asking follow up questions and checking my understanding) as I was aware that my response in those situations (e.g. scared) might be very different from that interviewee (e.g. excited). Many participants were inpatient and clinical staff did not sit in on interviews. This was to help ease any anxiety of participants that discussing details of their past violence might influence decisions about their progress and leave. Though it was made clear the limitations of confidentiality, participants were reassured that their interviews would not be shared with their clinical team. The impact of differences in gender, socio-economic status, and race were explicitly considered in discussions in supervision and with the LEAP throughout the interviews and analytic process.
